# Label-Free Electrochemical Detection of *S. mutans* Exploiting Commercially Fabricated Printed Circuit Board Sensing Electrodes

**DOI:** 10.3390/mi10090575

**Published:** 2019-08-30

**Authors:** Gorachand Dutta, Abdoulie A. Jallow, Debjani Paul, Despina Moschou

**Affiliations:** 1 Centre for Biosensors, Bioelectronics and Biodevices (C3Bio), Department of Electronic & Electrical Engineering, University of Bath, Bath BA2 7AY, UK; 2 School of Medical Science and Technology (SMST), Indian Institute of Technology Kharagpur, Kharagpur, West Bengal 721302, India; 3 Biosciences and Bioengineering Department, Indian Institute of Technology Bombay, Powai 400076, India

**Keywords:** electrochemical impedance spectroscopy, immunosensor, direct bacteria detection, lab-on-PCB (printed-circuit-board), *Streptococcus mutans* (*S. mutans*)

## Abstract

This paper reports for the first time printed-circuit-board (PCB)-based label-free electrochemical detection of bacteria. The demonstrated immunosensor was implemented on a PCB sensing platform which was designed and fabricated in a standard PCB manufacturing facility. Bacteria were directly captured on the PCB sensing surface using a specific, pre-immobilized antibody. Electrochemical impedance spectra (EIS) were recorded and used to extract the charge transfer resistance (R_ct_) value for the different bacteria concentrations under investigation. As a proof-of-concept, *Streptococcus mutans* (*S. mutans*) bacteria were quantified in a phosphate buffered saline (PBS) buffer, achieving a limit of detection of 10^3^ CFU/mL. Therefore, the proposed biosensor is an attractive candidate for the development of a simple and robust point-of-care diagnostic platform for bacteria identification, exhibiting good sensitivity, high selectivity, and excellent reproducibility.

## 1. Introduction

Bacteria comprise a ubiquitous type of microorganism, involved in numerous clinical, environmental, and industrial phenomena; indicative examples include infectious diseases, food and water safety, and insulin synthesis [1]. In all of the applications of this vast spectrum, there is the need to identify and quantify the presence of the bacteria with high sensitivity and specificity, ideally in real-time. The detection (i.e., quantification range, limit of detection) and usability (i.e., time to result, cost) requirements for each case vary, but nonetheless underpin in a similar manner all of them. For this reason, a multitude of research groups around the world have been proposing various bacterial detection technologies as alternatives to laborious standard protocols such as bacterial culture. For example, matrix-assisted laser desorption/ionization- time-of-flight MALDI-TOF mass spectrometry of culture supernatants, nucleic acid based methods (i.e., fluorescence in situ hybridization, DNA microarrays, real-time polymerase chain reaction (PCR). In all of the aforementioned cases though, the assay completion is laborious, requiring several sample preparation steps in order to achieve efficient quantification. Optical quantification methods, such as surface plasmon resonance imaging (SPRi) have also been reported, showing promising limits of detection in liquid culture media, but has also yet to be demonstrated in bacterial multiplication-free systems [2].

The importance of a minimal sample pre-processing, label-free detection system of high sensitivity becomes even more evident when we consider the incorporation of such systems in miniaturized, portable devices. The scientific and commercial consensus agrees that the future will be dominated by biosensors incorporated in such laboratory-on-a-chip (LoC) devices, offering the unique advantages of rapid reaction times, portability, high throughput, and automation. The bacterial biosensor is the heart of such systems, constructing the remaining parts around its requirements; the less laborious the detection protocol the more likely it is to have a cost-effective and practical device. To this end, electrochemical detection of whole bacteria [3], seem to be the ideal implementation to meet these requirements; they provide accurate quantification of the bacteria in the form of an electrical signal, require no cell culture, cell lysis, or DNA purification steps, have minimal and low power instrumentation requirements, and are easily miniaturized and integrated with sample handling microfluidic structures. A typical technology enabling the seamless integration of electrochemical biosensors and microfluidics is lab-on-PCB (Printed-Circuit-Board) technology [4]. Nonetheless, no lab-on-PCB system has been demonstrated so far featuring label-free electrochemical detection of whole bacteria. Hence, this is the scope of this work, demonstrating the first such PCB-based sensor, with *S. mutans* as an initial case study bacterium target.

Oral health is a major global crisis that cuts into government budgets across developed, developing, and underdeveloped communities [5,6]. The global burden of oral diseases remains very high with dental caries, periodontitis, and tooth loss being among the most common. Studies have shown that more than half of the world population has experienced dental caries and 90% of the disease goes undiagnosed [7]. This is largely due to the little attention that has been given to oral health; as a result, the large spectrum of diseases associated with it continue to pose major threats to human existence. This is evident by a recent (2018) United Nations (UN) General Assembly meeting that aimed to discuss the impact of non-communicable diseases but failed to make any mention of oral health [8]. Sadly though, oral health has been proven to share common risk factors and pathways with other non-communicable diseases. The majority of dental caries patients are the elderly and children under fifteen, with children from underprivileged backgrounds more likely to experience the disease than their counterparts from more privileged ones [9,10]. Dental caries is a multifactorial microbial disease that includes demineralization of tooth enamel, leading to cavity formation and sometimes tooth loss [11].

The healthy human oral cavity is made up of a complex microbiota of bacterial species that are necessary for the microbial balance in the gut and hence useful for its normal functioning. It is estimated that the number of micro-organisms in the gut ranges between 600–1200 species with the majority of these being commensal micro-organisms. The current paradigm implicates *S. mutans* as the initiator of the disease process with lactobacillus as the key player in its progression [12]. While it is unclear when and how *S. mutans* plays a role in the disease progression, many reports have implicated the species and hence have led to it being referred to as the “arch criminal of dental caries”. This is further supported by the many investigations that found *S. mutans* in biofilms of caries patients, mostly children under the age of twelve. While *S. mutans* is not a major component of the oral microbiota of normal individuals, it is able to initiate a bacterial succession under certain conditions leading to the replacement of commensal bacteria with more aggressive and virulent species. The characteristics of the species that enable it to dominate and initiate dental caries include the ability to: (i) survive under very acidic conditions in the mouth; (ii) the ability to store and utilise excess sugars; and (iii) store excess sugars and later use them [13]. 

In this work we have used commercially fabricated PCB board sensors and characterized systematically their electrochemical performance. We have also characterized the primary antibody for the selective antigen detection in a 96-well plate using the enzyme-linked immunosorbent assay (ELISA) method, and finally immobilized the thiolated-primary antibody on the PCB sensing surface followed by the concertation dependent antibody-antigen interactions and measurement of their subsequent impedance detection signal. For proof of principle, *S. mutans* was measured in buffer samples which could be detected down to 10^3^ CFU/mL. Several groups have demonstrated *S. mutans* detection using different biosensing methods [14,15,16,17,18,19,20]. While capable of rapid measurements, these sensors require complicated detection protocols which may not be ideally suited for point-of-care diagnosis. Here we report for the first time a PCB implemented label-free electrochemical *S. mutans* detection platform using a very simple detection protocol. *Streptococcus mutans* was detected on the PCB sensing surface using a specific antibody which was pre-immobilized on the PCB. Electrochemical impedance spectrometry (EIS) was used to record the charge transfer resistance (R_ct_) value with different *S. mutans* concentrations.

## 2. Experimental Methods

### 2.1. Materials 

Acetone, ethanol, ammonium hydroxide (NH_4_OH), hydrogen peroxide (H_2_O_2_), dimethyl sulfoxide (DMSO), potassium ferrocyanide (K_4_Fe(CN)_6_), potassium ferricyanide (K_3_Fe(CN)_6_), TMB (3,3’,5,5’-tetramethylbenzidine), and TMB stop solution were purchased from Sigma-Aldrich (Gillingham, UK). *Streptococcus mutans* strain MTCC890 was purchased from Microbial Type Culture Collection and Gene Bank (MTCC), Chandigarh, India. *Escherichia coli* (MG1655) was purchased from *E. Coli* Genetic Stock Center (Yale University, New Haven, CT, USA). Luria broth and the agar base were purchased from Himedia Labs (Bangalore, India). Primary anti-*Streptococcus mutans* antibody (ab31181) and goat anti-rabbit IgG H&L (HRP-ab205718) secondary antibody were purchased from Abcam (Cambridge, MA, USA). TYCSB (tryptone yeast extract cystine without sucrose and without bacitracin agar base) and Dulbecco’s phosphate buffered saline (PBS) were purchased from Himedia, India. The wash buffer was made up of 0.01 mol/L PBS (pH 7.4) mixed with 0.05% Tween-20. The blocking buffer was made up of 0.01 mol/L PBS (pH 7.4) mixed with 1% bovine serum albumin (BSA). USA. All other reagents were of analytical grade, and all aqueous solutions were prepared using 18.2 MΩ ultra-pure Milli-Q water.

### 2.2. Preparation of Bacterial Cultures

Brain heart infusion (BHI) broth was prepared by dissolving 3.7 g of the BHI base in 100 mL of distilled water. BHI plates were prepared by dissolving 5.2 g of the BHI agar in 100 mL distilled water. Luria broth was prepared by dissolving 2 g of Luria broth base in 100 mL of distilled water. Luria agar (LA) plates were prepared by 3.5 g of the LA powder to 100 mL of distilled water.

To prepare *Streptococcus mutans* cultures, 10 µL of the revived MTCC890 strain were first plated on BHI agar plates and allowed to grow overnight at 37 °C. From these plates, overnight liquid cultures were set up in fresh BHI broth. It was further diluted 400 times and incubated at 37 °C and 237 rpm for 48 h. 3 mL of the liquid culture was centrifuged at 15,000 rpm for 10 min to obtain a bacterial pellet. The pellet was washed thrice with warm 1X PBS (pH 7.4). The final washed pellet was diluted in 2 mL of 0.01 mol/L PBS (pH 7.4) and used for ELISA experiments. For the electrochemical detection, 6 mL of the primary culture was centrifuged and washed three times as previously described. The washed pellet was then diluted with 1 mL of 0.01 mol/L PBS (pH 7.4). We then serially diluted this bacterial suspension to prepare different concentrations of bacteria.

To determine the specificity of our sensor to *S. mutans*, we used *E. coli* (MG1655) as a negative control. We plated 10 µL of the revived *E. coli* on LA plates and incubated them overnight at 37 °C. From these plates, an overnight culture was set up. From the overnight culture, a concentration of 10^10^ CFU/mL was prepared in 2 mL of 0.01 mol/L PBS (pH 7.4). A high concentration of E. coli was chosen to test the binding specificity of the prepared electrodes. 

### 2.3. Thiolation of Anti-Streptococcus mutans Primary Antibody

The specificity of the anti-*Streptococcus mutans* polyclonal antibody was determined by indirect ELISA, as described in the supplementary material. The primary antibody was thiolated (-SH) as previously reported with minor modifications [21,22]. Briefly, 1 mL of 100 µg/mL anti-*Streptococcus mutans* IgG was incubated in a solution of Traut’s reagent in PBS containing 2 mM EDTA for 1 h at room temperature with gentle agitation. A 10-fold molar excess of Traut’s reagent per mol antibody was used to ensure full thiolation to the lysine side chains of IgG. Excess (unconjugated) Traut’s reagent was removed by centrifugation for 30 min at 10,000 rpm. Thiolated anti-*Streptococcus mutans* IgG was dissolved in 1 mL PBS (pH 7.4) and used immediately for sensor immobilization. 

### 2.4. Design of Printed Circuit Board Sensing Electrodes for S. Mutans Detection

The sensing electrodes were designed in PCB CAD software (version 17.1.9, Altium^®^) and commercially fabricated in a standard PCB manufacturing facility (Lyncolec Ltd, Poole, UK). The copper electrodes were electroplated with a hard-gold finish in order to exploit the pore-free deposition and low contact resistance achieved by this technique [23]. The gold-plated electrodes were exploited as working, counter, and reference electrodes and connected to a pocketstat (Ivium, Netherland) to record the signals. 

### 2.5. Sensor Fabrication

The gold-plated PCB electrodes were cleaned prior to anti-*Streptococcus mutans* IgG immobilization by ultrasonication in acetone, ethanol, and water, respectively for 15 min followed by 30 min ultrasonication in a solution containing 5:1:1 water, ammonium hydroxide (20%), and hydrogen peroxide (30%) [24,25]. Immobilization of the anti-*Streptococcus mutans* IgG to the working electrode was carried out by incubating 10 µg/mL of thiolated anti-*Streptococcus mutans* IgG solution for 1 h at room temperature, followed by thorough rinsing with PBS, and drying with purified N_2_ gas. A volume of 5 μL of the thiolated antibody was employed for spotting it on the surface via manual drop casting on the working electrode surface, assuring all liquid was in contact with the surfaces of the other two electrodes. To minimize nonspecific binding and enhance the stability of the immobilized antibody, 1% bovine serum albumin (BSA) dissolved in PBS solution was incubated on the antibody-immobilized electrode for 30 min at room temperature, followed by rinsing twice with PBS and drying with purified N_2_ gas. The prepared electrodes were stored at 4 °C [26,27,28,29].

### 2.6. Experimental Setup and Electrochemical Measurements

Cultures of *S. mutans* bacteria were serially diluted in PBS to obtain different concentrations, which were subsequently used for EIS measurements without any further processing. 50 µL of bacteria spiked in PBS was dispensed onto the sensor and incubated for 30 min at 4 °C, followed by thorough rinsing with PBS and drying with purified N_2_ gas. EIS measurements were performed immediately after bacteria incubation using a Helios electrochemical potentiostat (Ivium pocketstat, Netherlands), connecting the three PCB sensing electrodes through wires and a commercial Peripheral Component Interconnect (PCI) express connector. A micromachined Teflon tape was adhered on the board around the sensing area, in order to separate one electrochemical cell on the PCB from another, and locally confine the reagents. The impedance spectra were recorded using gold as counter and pseudo-reference electrodes at an open circuit potential (OCP) in the frequency range of 100 kHz to 0.5 Hz, with a 60 mV amplitude in 50 µL of 100 mM PBS (pH 7.4) containing 2 mM of the [Fe(CN)_6_]^3−/4−^ redox couple [30]. All electrochemical data were obtained at room temperature (25 °C). The cyclic voltammograms were performed in a three-electrode configuration with cycling the potential between −0.3 V and 0.4 V (scan rate: 0.05 V·s^−1^). 

## 3. Results and Discussion

### 3.1. Description of Label-Free S. mutans Detection Scheme

**Figure 1:**
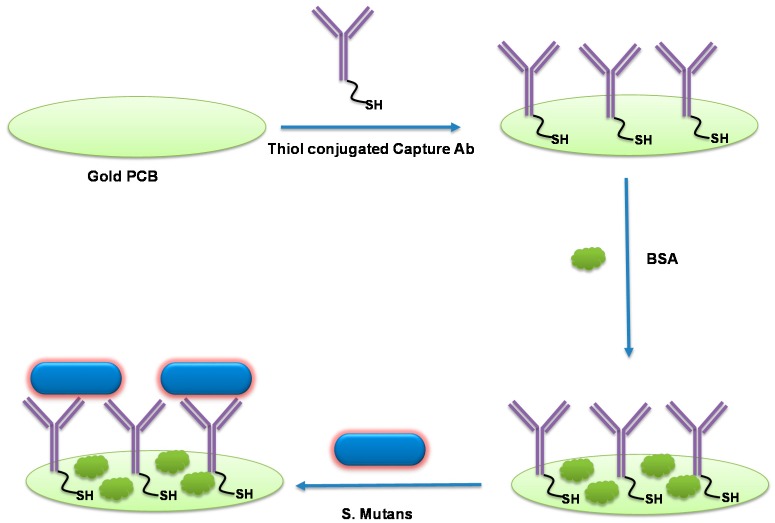
Schematic illustration of the label-free electrochemical *S. Mutans* detection scheme.

**Figure 2:**
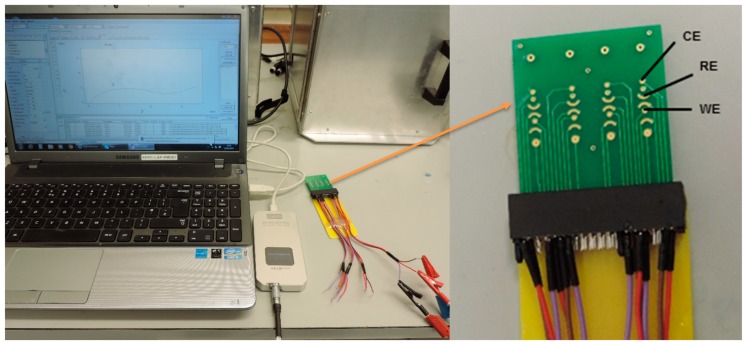
Illustration of the electrochemical impedance spectra (EIS) measurement setup using a pocketstat with a commercially fabricated printed-circuit-board (PCB). Inset shows a magnified view of a PCB in a three-electrode configuration.

**Figure 3:**
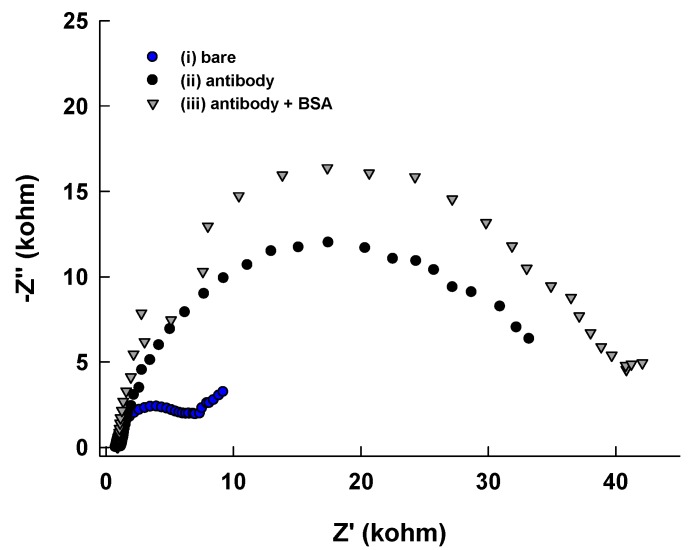
EIS responses of (i) bare PCB, (ii) antibody immobilized PCB, (iii) antibody and bovine serum albumin (BSA) modified PCB.

Figure 1 schematically illustrates the label-free *S. mutans* detection protocol on the PCB sensor surfaces. The thiolated capture anti-*Streptococcus mutans* IgG was immobilized effortlessly on the PCB surface because of the strong thiol-Au interaction. Non-specific binding on the sensor surface was minimized by BSA, allowing the functionalized sensor to selectively bind with *S. mutans.* The electrochemical impedance spectra were recorded at each step. The complete set up of the electrochemical detection of bacteria using pocketstat is shown in Figure 2. Figure 3 shows typical EIS spectra of bare PCB, antibody-immobilized PCB, and antibody- and BSA-modified PCB surfaces, respectively, in 0.1 M PBS containing 2 mM K_4_Fe(CN)_6_ and 2 mM K_3_Fe(CN)_6_. As seen in Figure 3, the charge transfer resistance (R_ct_) dramatically increases from 6.26 kΩ to 27.19 kΩ, confirming that the antibody was immobilized on the sensor surface. The R_ct_ value was further increased to 36.49 kΩ when BSA was incubated on the antibody-modified surface, confirming that BSA successfully blocked the unoccupied surface. The R_ct_ values for the ferri/ferrocyanide are increased with successive protein layer formation on the sensing surface because the protein coverage at the PCB Au surface hindered the rate of electron transfer to the Au, causing the observed increase in the R_ct_.

### 3.2. Preparation of S. mutans Cultures for Electrochemical Detection

In order to determine the concentration of bacteria in our electrochemical test samples, the primary culture was serially diluted by a factor of 10 and the colonies were counted. Ten microliters of each solution were plated on solid agar plates (TYCSB) and incubated for 48 h to allow the growth of bacteria colonies. From counting the number of colonies, the concentration of the primary culture was found to be approximately 1.68 × 10^9^ col/mL. 

### 3.3. Quantification of S. mutans Detection via Impedimetric Measurement

In order to validate the antibody binding to *S. mutans*, an indirect ELISA experiment was performed as described in the supplementary materials (Figures S1 and S2). The ELISA data confirmed that the primary antibody binds to our strain of *S. mutans* successfully. Hence, we proceeded with the functionalization of our sensor via immobilization of the antibodies on the working electrode surface, and recording of the respective EIS spectra.

**Figure 4:**
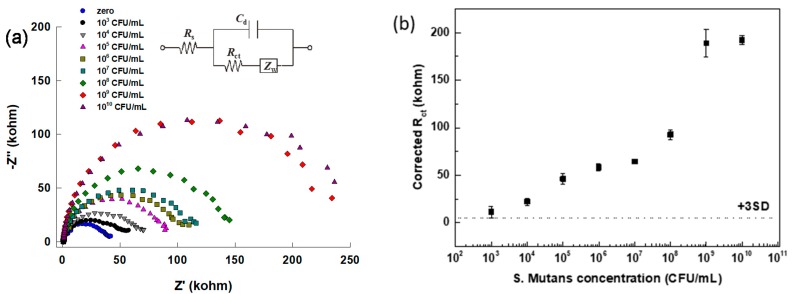
EIS responses of developed biosensor to detect different concentrations of *S. mutans* bacteria (**a**) the -Z” vs. Z’ ‘Nyquist plot’ with an inset showing the equivalent Randle’s circuit and (**b**) the extracted R_ct_ vs. bacterial concentration calibration curve.

The R_ct_ is the key parameter associated with the binding of the target to the capture antibody on the sensor surface, and hence, to the concentration of the *S. mutans* in the sample solutions. R_ct_ can be conveniently extracted from the EIS spectrum by either direct analysis of the spectrum or by fitting the spectrum to the Randles equivalent circuit (Figure 4a (inset)). The Randles equivalent circuit is composed of solution resistance (R_S_) in series with the parallel integration of the double-layer capacitance (C_dl_) and the charge transfer resistance (R_ct_) [31]. The Warburg impedance is known to be represented as the straight line with a 45° phase angle and is closely associated with the diffusion of the redox species in solution; if the Warburg impedance element is absent and the R_ct_ values of the sensor surfaces are significantly large, this implies that a significantly large amount of a charge transfer impeding material is found on the modified sensor. The R_ct_ of redox species is modulated by their inherent electron transfer rate and the presence of any charge transfer-impeding material on the electrode surface that the redox species must penetrate to reach the electrode surface, thus increasing the magnitude of the R_ct_. Impedimetric measurements were carried out after 60 µL of the redox couple was injected. Figure 4a shows representative R_ct_ values obtained from the PCB-implemented sensor surfaces treated with different concentration of *S. mutans* bacteria between 10^3^ CFU/mL and 10^10^ CFU/mL. Figure 4b presents the corresponding calibration plot of *S. mutans* detection, demonstrating the anticipated increase of R_ct_ with increasing *S. mutans* concentration. This assay exhibits a lower limit of detection (LOD) of 10^3^ CFU/mL. Three repetitions were done on the same PCB sensing surface. We observed a nonlinear behavior in the calibration curve. The signal was slowly increased with the lower *S. mutans* concentration, and the increment was much faster when the concentration of *S. mutans* was near 10^8^ CFU/mL. The protein layer coverage on the PCB Au surface is the key factor for the R_ct_ value change during the impedance measurement. We assume that the sudden jump behavior of the R_ct_ from 10^8^ to 10^9^ was because of more potential protein layer formation on the Au surface that effectively hindered the electron transfer rate to the Au. Finally, we observed a saturation of the impedance signal when the concentration of *S. mutans* reached near 10^10^ CFU/mL.

### 3.4. Specificity Study of the Developed Sensor

**Figure 5:**
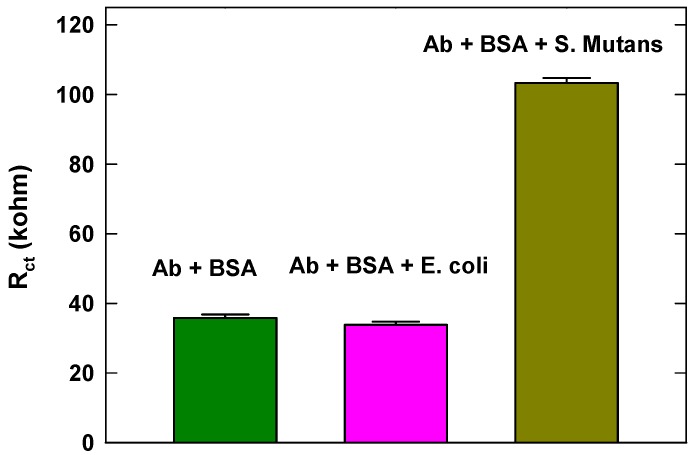
Specificity behavior of the developed biosensor in discriminating *S. mutans* against *E. coli*.

The specificity of this sensor was studied by performing measurements on PBS buffer samples spiked with *E. coli*, which is a Gram-negative, facultative anaerobic, rod-shaped, coliform bacterium of the genus Escherichia that is commonly found in the lower intestine of warm-blooded organisms. As shown in Figure 5, the R_ct_ value of 10^10^ CFU/mL *E. coli* modified sensor surface is similar to that generated from the non-spiked sample, which was used as a blank control. In contrast, the R_ct_ value from the sample containing 10^6^ CFU/mL *S. mutans* is significantly higher than that of *E. coli*, suggesting that our assay is highly specific to *S. mutans*.

## 4. Conclusions

In this paper we report the first PCB implemented label-free electrochemical bacterial biosensor. The thiolated capture antibody was successfully immobilized on a PCB sensing surface and we demonstrated that we can detect *S. mutans* selectively at a LOD of 10^3^ CFU/mL. This work suggests that it may be possible to exploit PCB biosensors to potentially profile multiple bacteria in completely untreated samples, such as saliva. The proposed PCB which is designed and fabricated in a standard PCB manufacturing facility shows promising bacteria detection results which could be particularly useful for point-of-care and field uses in developing countries. 

## Supplementary Materials

The following are available online at https://www.mdpi.com/2072-666X/10/9/575/s1, Figure S1: Selection and validation of the anti-*S. mutans* antibody by ELISA, Figure S2: Selection and validation of the anti-*S. mutans* antibody by ELISA.
